# Supplementary material for: Stakeholders’ attitudes to outdoor residual spraying technique for dengue control in Malaysia: A PLS-SEM approach
Source: PLoS Negl Trop Dis. 2021 Jun 29;15(6):e0009493. doi: 10.1371/journal.pntd.0009493 (PMC8274932; doi:10.1371/journal.pntd.0009493)
Supplement: Attachment — Submitted filename: Response to reviewers (Final ORS).doc [file pntd.0009493.s001.doc]

| **Reviewer’s Comments** | **Response** |
| --- | --- |
| **Methods**  **The objective of the study is stated namely to determine the factors that influence the attitudes toward the Outdoor Residual Spraying (ORS) and the moderating factor. However, it needs to be consistent whether the authors try to investigate stakeholder’s attitudes or Malaysian’s attitudes.**  **The study design needs to be improved. I think Figure 1 is not enough to present the conceptual research framework. It would be beneficial for the reader if the authors explained details regarding the exogenous variables, endogenous variables, a mediator variable, a moderator variable on the theoretical framework.**  **The population is clearly described and appropriate for the hypothesis being tested. The author also has calculated the statistical power and showed that the sample size of respondents is sufficient. The statistical analysis used to support conclusions were correct. No ethical approval was required in this study.** | ***Thank you for the comments.**  The authors have revised the objective of this study to investigate factors influencing stakeholders’ attitudes towards the Outdoor Residual Spraying (ORS) and religiosity as the moderating factor.  ***Thank you for the comments.**  The authors were agreed to improve the study design for the conceptual research framework by explained more about function of the variables (exogenous, endogenous, mediator and moderator) in the **Theoretical Framework and Hypotheses Development, Page 7 Paragraph 2 line 14 to 21 as follows:**  Attitude to ORS technique is the endogenous variable for the four exogenous variables: perceived benefits, perceived risks, trust in key players and attitude to nature versus materials. Perceived benefits and perceived risks acted as the mediating variables for the relationships between the variables: trust in key players and attitude to nature versus materials and attitude to ORS technique. Additionally, religiosity was proposed as the moderating factor, which affect the strength of the relationship between the exogenous variables and attitude to ORS technique.  **Page 7, last line until and Page 8, line 1 to 7 as follows:**  Fig 1 presents the conceptual research framework for the proposed relationships between the exogenous and endogenous variables, as well the role of the mediating and moderating variables. The hypotheses were developed based on the correlations results between the factors using the Pearson correlation method [26] (Table 1). The component-based approach of PLS-SEM (Partial Least Squares Structural Equation Modeling) of the SmartPLS software was used to test the hypotheses.  ***Thank you for the comments. Really appreciate it.** |
| **Results**  **The results are well presented but it needs to be improved. For example, in Table 2, the author may need to explain how they categorized high and moderate on the overall mean score of the factors. Some results need to be clarified (file attached). The tables are clearly presented.** | ***Thank you for the comments.**  The authors have explained the categorization of the overall mean score of the factors in the **Results and Discussions Section, Page 13 from line 20 to 25 and continue to Page 14, line 1 to 8 as follows:**  For ease of interpretation, the overall mean scores for all the variables have been categorized into three categories. If the mean scores were within the range of 1 to 3.00, they would be categorized as low. When the mean scores were in the range of 3.01 to 5.00, they would be classified as moderate, while mean scores of 5.01 to 7.00 were categorized as high. The overall mean scores for religiosity (mean score of 6.11), trust on key players (mean score of 5.51) and perceived benefits (mean score of 5.27) were in the high category. The stakeholders claimed that they were highly attached to their religion, were highly trusting of the key players and perceived the ORS technique as highly beneficial which was translated into a highly positive attitude to ORS technique (mean score of 5.28). On the other hand, the stakeholders were more inclined towards nature (mean score value were 3.92, below the mid-point of 4.0). Additionally, the stakeholders perceived the risks of the ORS technique as moderate (mean score of 3.30). |
| **Conclusions**  **The conclusion is supported by the data presented. The author clearly described the limitations of the analysis.** | ***Thank you for the comments. Really appreciate it.** |
| **Summary and General Comments**  **This study aims to present the factors that influence the stakeholder's attitudes toward the outdoor residual spraying technique. Overall, the manuscript is generally well-written. However, some points need to improved and clarified as follows:  Page 3: Under introduction, …serotypes-DENV-1, DEN-2,… Should be …serotypes-DENV-1, DENV-2,…  Page 6: Under theoretical framework, … “towards the ORS perceived benefit,….. Should be “towards the ORS, perceived benefit,….. (please put comma)  Page 7, paragraph 1: “Fig 1 presents   …..Malaysians’ attitude towards……” Should not be “ Fig 1 presents   …..stakeholder’s attitude towards……”?Please be consistent!**  **Page 7: under “Trust in Key Players”: …. Benefits and risks associated with GM product…” Please define the GM for the first time mentioned. Is that “Genetically Modified”?  Page 11: “In summary, the respondents were split into two groups: the public (n=197) and the scientists and implementers (n=202)” However, in the abstract, it is stated that “ …two stakeholder groups which consist of scientists, and the public ….”. Please be consistent in defining what the stakeholder is!  Page 12 under Results and Discussion: “ Table 2 present the overall mean scores of Malaysian attitudes…” Please be consistent!  Page 17 under Analysis of the Structural Model: “The value of the standardized root mean square (SRMR) …” Should not be ”The value of the standardized root mean square residual (SRMR) …”?**  **Page 18, the second paragraph: “The Q2 values for the perceived benefit (0.120), perceived risk (0.069), and attitudes towards the ORS approach (0.287)…” How did you get these numbers in bracket? Should not be: “The Q2 values for the perceived benefit (0.109), perceived risk (0.161), and attitudes towards the ORS approach (0.302)…”?**    **Page 21: ”… the VAF value was at 61.54%.”. How did you get 61.54?  Page 21, the last line: “…with the VAF value at 27.12%.”. How did you get 27.12%?**  **Page 23 under conclusion: “ The In conclusion…”. Please remove “The”.** | ***Thank you for the comment. Really appreciate it.** The authors have revised the sections suggested by the reviewer. …serotypes - DENV-1, DENV-2, DENV-3, and DENV-4, … the attitudes towards the ORS, perceived benefit, … Change the sentences and be consistent with the word ‘stakeholders’: Fig 1 presents the conceptual research framework for the proposed relationships between the exogenous and endogenous variables, as well the role of the mediating and moderating variables. The community does not directly assess the benefits and risks associated with genetically modified (GM) products, … In summary, the respondents were split into two groups: the public (n=197) and the scientists (n=202). Table 2 presents the overall mean scores of stakeholders’ attitudes towards the ORS and its predicting factors. The value of the standardized root mean square residual (SRMR) was … Thanks for correcting the values in the bracket with the table. The *Q^2^* value in the table is an incorrect value. Sorry for this mistake. The Q^2^ values for the perceived benefit (0.120), perceived risk (0.069), and attitudes towards the ORS approach (0.287) were greater than zero which confirmed that the model’s predictive relevance was adequate [65]. \| *R^2^* \| *Q^2^*; Attitude to ORS Technique \| 0.529 \| 0.287; Perceived Benefit \| 0.207 \| 0.120; Perceived Risk \| 0.106 \| 0.069 The author has explained how to get the VAF value. It was determined by dividing the direct effect with the total effect as suggested by Zhao et al. (2010), and if the value exceeds 20%, it indicates the mediating effect [70]. In conclusion, this research … |
